# Does walking after lumbar spinal surgery predict recovery of function at six months? Protocol for a prospective cohort study

**DOI:** 10.1186/s12891-016-1296-0

**Published:** 2016-11-14

**Authors:** Sarah Gilmore, Jodie A. McClelland, Megan Davidson

**Affiliations:** 1 St Vincent’s Private Hospital, Melbourne, Australia; 2 La Trobe University, Melbourne, Australia

**Keywords:** Physical activity, Physiotherapy, Rehabilitation, Lumbar fusion, Discectomy, Laminectomy

## Abstract

**Background:**

Physiotherapists are commonly involved in the management of patients immediately following lumbar spinal surgery. There is however, very little research to guide physiotherapy intervention in the acute post-operative period, and the advice provided to patients regarding post-operative walking and physical activity has been shown to be highly variable.

The primary aim of this research is to establish whether the amount of walking patients perform in the week following lumbar spinal surgery predicts improvement in function at 6 months.

**Methods:**

This study will be a prospective cohort study design, with a projected sample size of 250 participants. Patients undergoing surgery for the management of a disc prolapse, degenerative disc disease, lumbar spinal stenosis and/or degenerative spondylolysthesis will be invited to participate in this study.

Outcome measurement will take place pre-operatively and at six months post-operatively. The primary outcome variable will be self-reported function, measured using the Modified Oswestry Disability Questionnaire and the physical component summary of the SF-36.

Each participant will be fitted with an activPAL3 accelerometer to be worn for the first seven post-operative days. This accelerometer will record time spent in active versus sedentary postures, step count and time spent walking. Multivariable logistic regression analysis will be used to investigate the relationship between the total time spent walking over the first seven post-operative days, and outcome at six months.

**Discussion:**

The results from this research will help to guide patient management during the inpatient phase, by identifying patients who are at risk of poorer outcome due to limited walking time. These patients may benefit from ongoing rehabilitation and outpatient physiotherapy services. This information will also provide a foundation for further research into interventions designed to optimise post-operative activity.

**Trial registration:**

ACTRN12616000747426, retrospectively registered 7th June 2016.

## Background

Low back pain is the leading cause of disability worldwide, with both the rate of low back pain and the associated burden of disability expected to increase as the global population ages [1]. Clinical guidelines consistently recommend conservative management of low back pain, however surgical intervention may be indicated where conservative management is not successful [2]. While there is a growing body of evidence to support surgical management of low back conditions [3–5], up to 40 % of patients continue to report no or minimal improvement in post-operative function [6]. This figure highlights the need for evidence based, effective rehabilitation programs designed to optimise post-operative recovery.

Physiotherapy is a common component of the management of patients undergoing lumbar spinal surgery at the majority of hospitals in Australia and the UK [7–9]. The goals of physiotherapy intervention during the inpatient admission consistently focus on achieving independence with mobility and functional tasks, and providing advice and education about post-operative walking and physical activity [7–9]. The specific interventions required to achieve these goals however, are poorly understood. There is very little research to guide physiotherapy intervention in the acute post-operative period [10, 11], with considerable variability in the advice provided to patients regarding post-operative walking and general activity [7–9, 12].

There are a growing number of studies aimed at identifying factors that predict outcome following lumbar spinal surgery. These include patient demographics (age [13], smoking status [14–16]), co-morbidities (diabetes [14, 16, 17], obesity [14, 18]), impairments (pain and sensory changes [14]), and psycho-social factors (depression [19, 20], fear avoidance [21]). These factors play an important role in selecting patients appropriate for surgical intervention. There is however, limited scope to modify any of these variables particularly in the acute post-operative phase, to influence patient rehabilitation or functional recovery.

Clinical guidelines for the management of low back pain consistently recommend staying active [2] with evidence suggesting that maintaining an active lifestyle leads to reduced pain and improved functional outcome [22]. It is not known whether this positive association can be translated into the post-surgical population, or more specifically, whether people who participate in more physical activity have better outcomes following surgery. In other post-surgical populations, evidence suggests that early and increased walking improves functional outcome and reduces the rate of post-operative complications [23]. It is therefore likely that increased walking immediately following lumbar spinal surgery leads to similarly improved outcomes.

Accelerometers are becoming an increasing popular method of quantifying physical activity, and have been shown to provide an accurate measure of step count, time spent walking and distance walked. [24–26] Two recent studies have used accelerometry to describe the improvement in physical activity following lumbar spinal surgery. Mobbs et al. [27] reported a significant improvement in step count and distance walked three months after lumbar spinal surgery, and Schulte et al. [28] reported a significant improvement in step count three months after surgery for lumbar stenosis. There are however, no known studies describing walking immediately following spinal surgery, or how the amount of walking in this period impacts longer-term functional recovery.

We know that gait assessment, and advice regarding walking and return to physical activity already forms a key component of physiotherapy intervention immediately following lumbar surgery. Establishing how much walking patients currently do, and how walking in the period immediately following surgery impacts longer term functional outcome will enable physiotherapists to individualise patient management to achieve optimal outcomes. It will also help to identify patients who are unable to achieve the minimum amount of walking associated with improved outcomes, and who may therefore require more intensive rehabilitation programmes.

## Method

### Aims

This research will investigate the relationship between the time spent walking in the week following lumbar surgery, and recovery of physical function. The primary aim is to establish whether the amount of walking patients do in the week following lumbar spinal surgery predicts improvement in function at six months. The data collected will also be used to generate an activity profile to describe the overall patterns of activity in the first post-operative week, and to identify factors that may influence the amount of activity patients undertake.

The research questions are:

1. Does the amount of time spent walking during the week after lumbar spinal surgery predict improvement in function at six months?
2. What proportion of time do patients spend performing active tasks (standing and walking) compared to sedentary behaviour (sitting and lying) in the week following lumbar spinal surgery?
3. Is the amount of time spent walking in the first post-operative week influenced by restricted pre or post operative physical function, post-operative pain or complications, or the need for supervision while walking?

### Design

This study will be a prospective cohort study design. Ethics approval from the St Vincent’s Hospital Melbourne Human Research Ethics Committee’s has been obtained (Reference: LRR 098/15).

### Participants

Participants will be recruited from St Vincent’s Private Hospital, Fitzroy (SVPHF) over a six-month period. All patients aged 18 years and older undergoing surgery for the management of a disc prolapse, degenerative disc disease, lumbar spinal stenosis and/or degenerative spondylolysthesis will be invited to participate. There will be no restriction on the nature or duration of pre-operative symptoms. Patients will be excluded if they are undergoing surgery for the management of lumbar fractures or tumours, have a history of dementia or cognitive impairment or an inability to provide informed consent, or have a history of a neurological or musculoskeletal condition resulting in progressive impairment of physical function (for example, multiple sclerosis or rheumatoid arthritis). In cases where surgery includes both lumbar and thoracic vertebrae, patients will be eligible if 50 % or more of the vertebra involved are in the lumbar region.

### Study procedure

#### Recruitment and sample size

The recommended sample size for logistic regression analysis is a minimum of 5 outcome events per predictor variable (EPV) [29]. The maximum number of variables to be analysed in this study is 15 giving a minimum sample size of 150. Based on expected admissions to the treating institution over the recruitment period, it is expected that this will provide an estimated recruitment pool of 300 eligible participants and sample size of approximately 250 participants. This proposed sample size allows for variation in EVP, missing data and participants lost to follow-up.

Patients undergoing lumbar spinal surgery will be identified from the surgical lists. All eligible patients will be provided with an information pack containing a participant information statement, consent form, withdrawal of consent form and baseline outcome measures. The research physiotherapist will contact potential participants during the week prior to surgery to confirm eligibility and respond to any questions. Written consent will be obtained from all participants. Consent forms and baseline outcome measures will be collected from participants following admission to hospital. Demographic data and information about the surgical procedure will be collected from the participant and patient records during admission.

Following surgery, all participants will be fitted with an activPAL3 accelerometer (PAL technologies) to be worn for the first seven post-operative days, commencing the morning after the day of surgery. As per the manufacturer instructions, the accelerometer will be fixed to the upper thigh using a waterproof transparent Tegaderm dressing. This can then remain in place for the seven-day monitoring period, with a daily review to check for discomfort or irritation. This method of fixing the accelerometer to the thigh eliminates the need for participants to remove and apply the monitoring device for showering and sleeping, it does not require participants to wear a waist band or belt that would sit across the site of the lumbar surgery.

Where participants are discharged during the seven-day monitoring period they will be provided with information regarding the use and removal of the accelerometer, and a reply paid envelope to return the accelerometer to the researcher.

#### Six-month follow-up

At 6 months following surgery participants will be sent follow-up outcome measures, to be returned to the researchers by mail.

All participants will receive routine inpatient physiotherapy management. As part of routine management participants are encouraged to walk at least 3 times daily over a comfortable distance, increasing both the frequency and distance as able. An exercise program is also commenced within the first 3 days following surgery, and includes activation of deep core muscles in a supine position, exercises to gently increase range of motion including lumbar rotation and neural slides, and lower limb strengthening. A single researcher will conduct all participant communication, measurement, data collection and application of accelerometers.

### Outcome measures

An outcome assessment timeline and summary of assessment tools used is provided in Table 1.

**Table 1:** Timeline of outcome assessment

Pre-operative (baseline)	
Baseline demographics:	Age, sex, smoking status, height, weight, diabetic history
History of back/leg symptoms:	Duration of symptoms, neurological deficit
Physical function:	ODQ, SF-36
Pain:	Back and leg pain (NRPS)
Physical activity:	IPAQ-SF
Psychological status:	Depression (PHQ-9), anxiety (GAD-7)
Post-operative (days 1–7):	
Surgical procedure:	Decompression, discectomy, fusion; single or multi-level
Accelerometry:	Time spent walking, step count, time spent in sedentary and active postures, number of walks
Pain:	Back, leg and wound pain (NRPS)
Supervised walking:	Patient/physiotherapist reported
Post-operative complications:	Complications requiring medical intervention and/or limit walking time
Post-operative (6 months):	
Physical function:	ODQ, SF-36
Patient satisfaction:	5 point likert scale
Post-operative complications:	Complications requiring medical intervention

#### Dependent variable

The primary dependent variable will be self-reported function. This will be measured using a Modified Oswestry Disability Questionnaire (ODQ) [30] and the physical component summary (PCS) of the SF-36 (version 2) [31, 32]. Back and leg pain intensity using the numeric pain rating scale (0–10) (NPRS) [33] will be recorded as secondary dependent variables. The ODQ, SF-36 and the NPRS are all validated and recommended for use within the back pain population [34], and are widely used in spinal surgery research. The modified ODQ substitutes kilometres for miles to estimate walking distance, and has been validated in the Australian population [30]. The relationship between post-operative walking and each of the four dependent variables will be analysed individually.

#### Independent (predictor) variable

The predictor variable to be used in data analysis will be the total time spent walking (recorded as total time performing stepping activity) over the first seven post-operative days. This data will be recorded using an activPAL3 accelerometer and downloaded using software provided by PALtechnologies. The activPAL3 accelerometers provide a user-friendly measure of posture and walking with robust evidence for validity and sufficient reproducibility for this study [24–26]. As the activPAL3 accelerometer is worn on the thigh, the accuracy of the monitor is not reliant on arm swing and is not expected to vary if participants are using a gait aid.

#### Independent (confounding) variables

Data will be collected for variables where there is either evidence to suggest they influence outcome following lumbar spinal surgery, or there is or strong theoretical rational for their inclusion. This will include age, gender, current smoking status [14, 15], obesity [14, 18], diabetes [14, 16, 17], depression [19, 20], duration of pain/symptoms [35], neurological deficit [14], anxiety, pre-operative activity, pre-operative mobility, surgical procedure, multi-level surgery, and pre-operative function.

Depression will be assessed using the Patient Health Questionnaire Depression Scale (PHQ-9), using a cut off score of ≥10 [36]. The PHQ-9 is a short, valid and reliable measure of depression, and has been recommended for use within the chronic back pain population [37]. Anxiety will be scored using the Generalised Anxiety Disorder 7-item Scale (GAD-7), using a cut off score of ≥10 [38]. The GAD-7 is a brief, seven question scale used to screen for, and assess the severity of anxiety. It has been validated in the general population [38]. Neurological deficit will be assessed by asking the participant if he/she is aware of any changes to the sensation or strength in their affected lower limb. This level of information is deemed appropriate for this study, as any sensory or motor deficit that the participant is unaware of is unlikely to have an impact on functional ability. Pre-operative activity will be assessed using the International Physical Activity Questionnaire Short Form (IPAQ-SF) [39, 40]. The IPAQ-SF is a brief, reliable seven item questionnaire that requires participants to report on physical activity undertaken over the previous seven day period [39, 40]. Physical activity is categorised into low, moderate and high activity levels. As it is expected that the majority of participants will report either low or moderate activity levels, the two categories of low and moderate/high activity levels will be used for data analysis. Baseline ODQ score will be used as a measure of pre-operative function. Pre-operative mobility will be categorised based on the response to Section 4 (Walking) on the ODQ. Participants will be categorised as having limited mobility if they score 3 or more - unable to walk more than 500 m, require a walking aid, or are confined to bed.

#### Post-operative activity profile

A seven day activity profile summarising the amount of time spent in sedentary and active postures, time spent walking, a step count, and the number of walks will be generated using the data downloaded from the activPAL3 accelerometers.

#### Factors associated with time spent walking immediately following surgery

Additional data will be collected on a daily basis regarding post-operative back, leg and wound pain intensity (NRPS), the need for supervision while walking, and surgical/medical complications. Reported complications will include events where the participant requires medical intervention in addition to routine post-operative care, and events that limit activity (for example, symptomatic hypotension, analgesia-induced nausea). This information will be sourced from the patient, ward staff, and patient notes. In cases where participants are discharged from the inpatient facility prior to completing the monitoring period they will be asked to record this information daily, which will then be returned to the researchers with the accelerometer following the completion of the monitoring period.

At six months, participants will be asked to report on overall satisfaction with the results of the surgery, scored on a five point likert scale. They will also be asked to describe any post-operative complications experienced after the seven day monitoring period. This will include any event directly related to the surgical procedure requiring medical intervention in addition to routine follow-up care.

### Data analysis

Baseline demographics of the study population will be analysed using descriptive statistics.

#### Relationship between time spent walking and outcome

Multivariable logistic regression analysis will be used to investigate the relationship between the time spent walking in the first week after surgery and outcome at six months using SPSS software. The dependent variable in the logistic regression model will be the change in score on the ODQ, SF-36 (PCS), NRPS (back) and NRPS (leg) between baseline and six months. The change in scores on the ODQ, SF-36 (PCS) and NRPS will be dichotomised based on the threshold required to demonstrate a substantial clinical benefit.

Thresholds required to meet the minimum clinically important difference (MCID) have been identified for the ODQ, the SF-36 (PCS) and the NPRS [41] (Table 2). In addition, thresholds required to obtain a substantial clinical benefit (SCB), where patients report a major improvement in symptoms, have been defined for the ODQ, SF-36 (PCS) and NRPS in the lumbar fusion population [42] (Table 2). For the purpose of this study it has been assumed that SCB following lumbar fusion surgery provides a fair estimate of substantial clinical benefit following lumbar surgery in general. These thresholds allow the change in scores to be categorised as minimal improvement (below MCID threshold), moderate improvement (above MCID threshold, below SCB threshold) and substantial improvement (SCB threshold or greater).

**Table 2:** MCID and SCB thresholds

Outcome measurement tool	MCID (points change)	SCB (points change)
ODQ	10	18.8
SF-36 (PCS)	4.9	6.2
NPRS (back)	2	2.5
NRPS (leg)	2	2.5

As all participants in this study are undergoing surgery with the aim of improving pain and physical function, it expected that the majority of participants in this study will report an improvement in scores above the MCID threshold. Therefore the SCB threshold will be used to analyse the impact of walking time on outcome (minimal/moderate improvement compared to substantial improvement). For each of the four dependent variables a multivariable logistic regression model will be generated comparing participant outcome relative to the SCB threshold.

With the exception of pre-operative function and surgical procedure, all confounding variables will be dichotomised (Table 3).

**Table 3:** List of independent variables and classification for statistical analysis

	Classification				
1. Post-operative activity	Time spent walking (minutes)				
2. Age	1. < 65 years	2. ≥ 65 years			
3. Gender	1. Male	2. Female			
4. Current smoking status	1. Non-smoker	2. Smoker			
5. Obesity	1. BMI < 30	2. BMI ≥ 30			
6. Diabetes	1. Not diabetic	2. Diabetic			
7. Depression	1. PHQ-9 < 10	2. PHQ-9 ≥ 10			
8. Anxiety	1. GAD-7 < 10	2. GAD-7 ≥ 10			
9. Duration of pain/symptoms	1. < median (months)	2. ≥ median (months)			
10. Neurological deficit	1. No deficit	2. Neurological deficit			
11. Pre-operative activity	1. IPAQ-SF - Mod/high	2. IPAQ-SF - low			
12. Pre-operative mobility	1. ODQ (section 4) < 3	2. ODQ (section 4) ≥ 3			
13. Surgical procedure	1. Decompression	2. Disc surgery	3. Fusion		
14. Multi-level surgery	1. Single level surgery	2. Multi-level surgery			
15. Preoperative function (ODQ%)	1. 0–20	2. 21–40	3. 41–60	4. 61–80	5. 81–100

Bivariate analyses will be carried out to assess the association between each confounding variable and each outcome measure. The variables that have an independent association with outcome will be included in the final regression model. Analysis of correlation between the independent variables will be carried out to assess for multicollinearity. Where variables are highly correlated, either the more relevant variable or the variable with the more complete data set will be retained in the final regression model [43].

Missing independent variable data will be managed based on the quantity of missing data per participant and per variable. In the case of missing or incomplete accelerometry data, participants will be removed from analysis where three or more days of recordings are missing. In the case of one to two days of missing data, the time spent walking each day will be estimated based on the mean daily walking time of the total sample population. Comparisons will be made between the characteristics of participants with and without missing data to ensure minimisation of bias [43]. The underlying assumptions of the model will be assessed using the SPSS software “goodness of fit” testing.

Bivariate analysis will be used to examine the association between post-operative walking time, and both patient satisfaction and post-operative complications as reported at six months.

#### Post-operative activity profile

Descriptive statistics will be used to analyse post-operative activity.

#### Factors associated with post-operative walking time

Bivariate analysis will be used to examine the association between the total time spent walking, and pre-operative mobility, function and physical activity, post-operative back, leg and wound pain, post-operative complications and the need for supervision while walking.

## Discussion

The findings from this research will assist physiotherapists to identify patients who are expected to have either very good or poor post-operative outcomes based on their activity levels immediately following surgery, and inform physiotherapists about whether increasing activity should be a focus of post-operative rehabilitation. This will help to guide patient management during the inpatient phase, by identifying patients who are at risk of poorer outcome due to limited walking time. These patients may benefit from ongoing rehabilitation and outpatient physiotherapy services. This information will also provide a foundation for further research into interventions designed to optimise post-operative activity such as patient education resources, improved monitoring and management of post-operative pain, or the provision of additional physiotherapy sessions for patients who require supervision while walking.

## Abbreviations

EVP: Events per predictor variable
GAD-7: Generalised Anxiety Disorder 7-item Scale
IPAQ-SF: International Physical Activity Questionnaire Short Form
MCID: Minimum clinically important difference In addition
NPRS: Numeric pain rating scale
ODQ: Oswestry Disability Questionnaire
PHQ-9: Patient Health Questionnaire Depression Scale
SCB: Substantial clinical benefit
SF-36 (PCS): SF-36 physical component summary
SVPHF: St Vincent’s Private Hospital, Fitzroy
